# Book Reviews

**Published:** 1910-07

**Authors:** 


### BOOK REVIEWS.

PROGRESSIVE MEDICINE. A Quarterly Digest of Advances, Discoveries, and Improvements in the Medical and Surgical Sciences, Edited by Hobart Amory Hare, M. D., and Leighton F. Appleman, M. D. Vol. II., June, 1910. Lea & Febiger, Publishers, Philadelphia.

The first 71 pages of this volume are taken up by William B. Coley, M. D., in a most interesting digest of the recent literature of Hernia. Edward M. Foote, M. D., takes up Abdominal Surgery exclusive of Surgery, and covers the advances recently made in this field of surgery. The subject of Gynecology is then ably reviewed by Jno. G. Clark, M. D. Diseases of the blood, diathetic and metabolic diseases of the thyroid gland, nutrition and the lymphatic system are covered by Alfred Stengel, M. D. Jackson completes the volume with Ophthalmology. The world-wide reputation of these men and the unusual experience in the respective fields of their endeavor lend weight to the digests of the advances, etc., they review and thus make of *Progressive Medicine,* as we have said on former occasions, the best of journals of this type.

---

DUODENAL ULCER. By B. G. A. Moyniham, M. S., (London) F. R. C. S., Senior Assistant Surgeon at Leeds General Infirmary, England. Octavo of 379 pages, illustrated. Philadelphia and London: W. B. Saunders Company, 1910. Cloth, $4.00 net; half morocco, $5.50 net.

Seldom has the reviewer had the pleasure of reading a more original or entertaining book than Moynihan's on Duodenal Ulcer. One unfamiliar with the subject would be inclined to believe that in the diagnosis of such a lesion much doubt and uncertainty would exist; and yet such is not the case. The author seems most confident of his diagnosis when made from the anamnesis alone, and even then the important symptoms are those the physician recognizes as common complaints of chronic "dispeptics." The time is certainly not far distant when patients will be operated upon for indigestion just as quickly as for stone in the bladder, and with results just as certain. And it will be such monographs as this that will develop such procedures.

Among the many and great developments of modern abdominal surgery, few of the problems unravelled have been more interesting that those concerned with duodenal ulcer. Formerly it was thought to be a rare disease, now it is known as a common one which may be discovered without difficulty by the trained clinican. To Monyniham and the Mayos we owe nearly all the credit for the advances. Every physician should have this book and discontinue indiscriminate "drugging" of patients for the interminable "dispepsia" and gastric "neuroses."

---

MEDICAL ELECTRICITY AND RONTGEN RAYS. By Sinclair Tousey, A. M., M. D., Consulting Surgeon to St. Bartholomew's Clinic, New York City. Octavo of 1116 pages, with 750 illustrations, 16 in colors. Philadelphia and London: W. B. Saunders Company, 1910. Cloth, $7.00 net; half morocco, $8.50 net.

It seems that Tousey has overlooked no useful or recognized

Page(s) missing

